# Chronotype and emotion processing: a pilot study testing timing of online cognitive bias modification training

**DOI:** 10.1136/bmjment-2024-301045

**Published:** 2024-07-02

**Authors:** Charlotte M Crisp, Emily Mooney, Mohini Howlader, Joel Stoddard, Ian Penton-Voak

**Affiliations:** 1 School of Psychological Science, University of Bristol, Bristol, UK; 2 NIHR Bristol Biomedical Research Centre, Bristol, University Hospitals Bristol and Weston NHS Foundation Trust UK, UK; 3 School of Medicine, University of Colorado Anschutz Medical Campus, Aurora, Colorado, USA

**Keywords:** Cross-Sectional Studies, Data Interpretation, Statistical, Depression & mood disorders, Sleep

## Abstract

**Background:**

Circadian rhythms influence cognitive performance which peaks in the morning for early chronotypes and evening for late chronotypes. It is unknown whether cognitive interventions are susceptible to such synchrony effects and could be optimised at certain times-of-day.

**Objective:**

A pilot study testing whether the effectiveness of cognitive bias modification (CBM) for facial emotion processing was improved when delivered at a time-of-day that was synchronised to chronotype.

**Methods:**

173 healthy young adults (aged 18–25) with an early or late chronotype completed one online session of CBM training in either the morning (06:00 hours to 10:00 hours) or evening (18:00 hours to 22:00 hours).

**Findings:**

Moderate evidence that participants learnt better (higher post-training balance point) when they completed CBM training in the synchronous (evening for late chronotypes, morning for early chronotypes) compared with asynchronous (morning for late chronotypes, evening for early chronotypes) condition, controlling for pre-training balance point, sleep quality and negative affect. There was also a group×condition interaction where late chronotypes learnt faster and more effectively in synchronous versus asynchronous conditions.

**Conclusions:**

Preliminary evidence that synchrony effects apply to this psychological intervention. Tailoring the delivery timing of CBM training to chronotype may optimise its effectiveness. This may be particularly important for late chronotypes who were less able to adapt to non-optimal times-of-day, possibly because they experience more social jetlag.

**Clinical implications:**

To consider delivery timing of CBM training when administering to early and late chronotypes. This may generalise to other psychological interventions and be relevant for online interventions where the timing can be flexible.

**WHAT IS ALREADY KNOWN ON THE TOPIC**

- Synchrony effects (better performance at a time-of-day that is aligned to individual chronotype) are observed for a range of cognitive tasks. However, it is unknown if synchrony effects apply to psychological interventions that require similar cognitions, alertness and engagement.

**WHAT THE STUDY ADDS**

- Young adults who are allocated to complete an online psychological therapy at a synchronous time-of-day (eg, evening for late chronotypes, morning for early chronotypes) learn better than if they are allocated to an asynchronous time-of-day, particularly for late chronotypes. This suggests the delivery timing of psychological interventions is important as it changes effectiveness.

**HOW THIS STUDY MIGHT AFFECT RESEARCH, PRACTICE OR POLICY**

- Further evidence is needed but the findings could inform advice when prescribing psychological interventions and inform research methodology in psychological intervention studies.

## Background

Nearly one-third of young people in the UK aged 16–24 report symptoms of depression.^1^ An important risk factor for depression is chronotype. Chronotype is a marker of circadian physiology and refers to differences in diurnal preference where late chronotypes (‘night owls’) go to bed late, wake up late and reach their peak performance late in the day, whereas early chronotypes (‘morning larks’) are the opposite. Chronotype is determined by genetics, light exposure and age^2^ where more young people are late chronotypes (15–20% of adults aged 18–25).^3^ Moreover, young late chronotypes are more likely to develop depression,^4^ which may be because they experience more ‘social jetlag’—a misalignment between an individual’s biological clock and social demands.^5^ Late chronotypes typically accumulate sleep debt on weekdays (due to early start times for school/work) and compensate by sleeping for longer on the weekend. Social jetlag may act as a chronic stressor that interacts with other factors (eg, genetics) to increase the risk for depression. Therefore, late chronotype is over-represented in young people and is a risk factor for depression, so this is a key vulnerable population to focus tailoring interventions for.

Previous research has shown that healthy, late chronotypes display negative cognitive biases. For example, they show increased recognition of negative facial expressions,^6^ and structural and functional differences in brain networks responsible for emotion processing.^7^ These negative processing biases are similar to those reported in patients with depression and are proposed to be on the causal pathway since negative biases occur before the onset of depression^8^ and positive biases emerge after antidepressant administration but before clinical improvement in mood.^9^ It is proposed that negative cognitive biases could be cognitive vulnerabilities that occur in response to social jetlag and increase the risk for depression in late chronotypes. Studies of low mood and depression have shown that cognitive bias modification (CBM) for facial emotion processing is an intervention that is effective at changing negative processing biases by shifting appraisals of ambiguous facial expressions away from negative (sad) and toward positive (happy) emotions through training.^10 11^ CBM training is proposed to establish a ‘virtuous cycle’ whereby altering the perception of facial expressions leads to more positive social behaviours which are reciprocated and reinforced by continued social interactions.^12^ Therefore, CBM training could be relevant for late chronotype individuals to boost positive cognitions.

Chronotype might be an important consideration for tailoring interventions because circadian rhythms influence alertness, mood and cognitive function. For example, performance on cognitive tasks that require sustained attention is improved when completed at an ‘optimal’ time of day according to chronotype (evening for late chronotypes, morning for early chronotypes).^13^ This is according to circadian patterns of arousal and is known as the ‘synchrony effect’.^14^ For example, Hahn *et al* 13 reported that adolescents (aged 11–14) performed better on executive function tasks (eg, Iowa Gambling Task, working memory task) when tested at their optimal time of day according to chronotype, that is, afternoon for late chronotypes and morning for early chronotypes.^13^ However, it is unknown whether psychological interventions (including CBM for facial emotion processing) that require similar executive functions could be optimised when delivered at a time-of-day that is tailored to chronotype. It could be that optimising delivery timing may allow young people to employ more cognitive resources, learn better and engage more with the intervention, which in turn would improve its effectiveness. This could be translated to other psychological interventions.

### Objective

A pilot study to understand whether the effectiveness of CBM for facial emotion processing is improved when delivered at a time-of-day aligned to chronotype. Healthy, young adults (aged 18–25) with either an early or late chronotype completed CBM training online in either the morning (06:00 hours to 10:00 hours) or evening (18:00 hours to 22:00 hours) session. We predicted that the effectiveness of CBM for shifting interpretation of emotional facial expressions (learning rate and post-training balance point) would be optimised when delivered in a ‘synchronous’ condition (evening session for late chronotypes and morning session for early chronotypes) compared with an ‘asynchronous’ condition (morning session for late chronotypes and evening session for early chronotypes), and this effect would be greater in the late chronotypes compared with early chronotypes (group×condition interaction). We also predicted that online CBM would have a greater effect on improving positive and negative effects in late chronotypes compared with early chronotypes.

## Methods

### Study design

**Figure 1:**
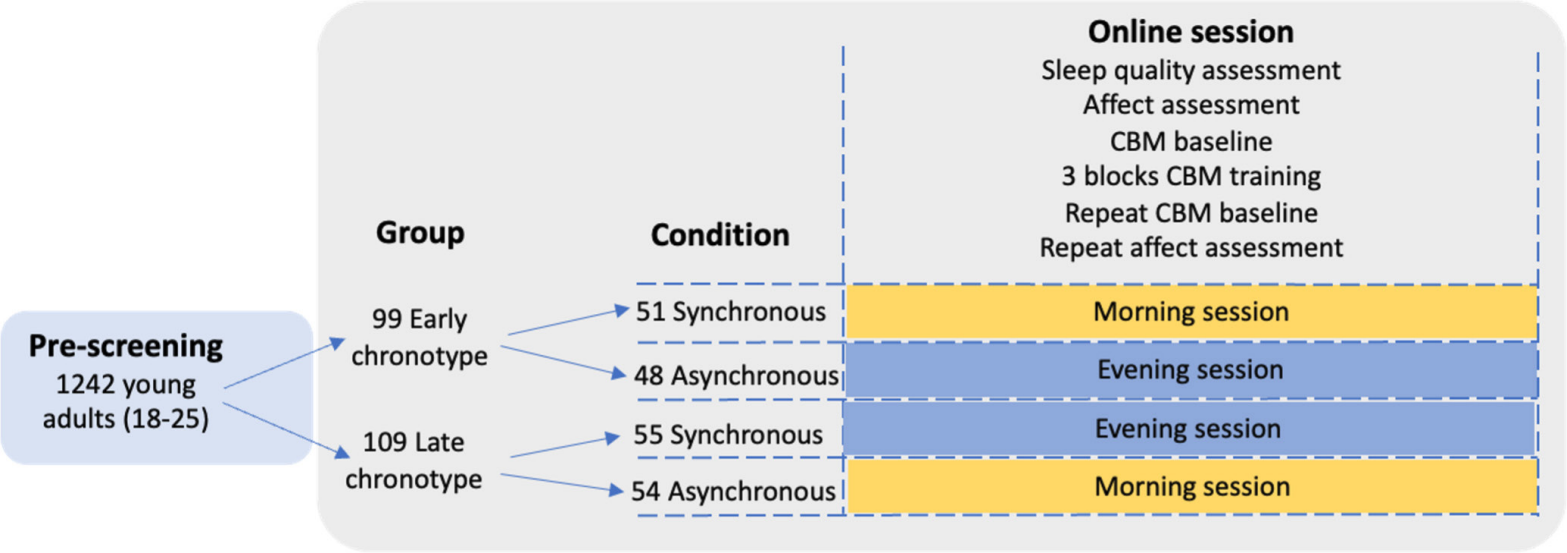
Study design. Details of assessments carried out during the main study session using a four-parallel group design. CBM, cognitive bias modification.

The study design can be viewed in figure 1. Young adults were pre-screened using the online recruitment platform Prolific (https://www.prolific.co) and Qualtrics (https://www.qualtrics.com/uk/). Prolific hosts a large pool of diverse participants to recruit from and was used to avoid recruiting a student-only sample and improve generalisability. Adults with a late or early chronotype were then recruited to the main study through Prolific which was conducted remotely and online using Gorilla (https://gorilla.sc/). Half of each chronotype group was allocated to CBM training in a morning (06:00 hours to 10:00 hours) or evening (18:00 hours to 22:00 hours) session, that is, in a synchronous (morning for early chronotypes, evening for late chronotypes) or asynchronous (morning for late chronotypes, evening for early chronotypes) condition (2×2 design). An effort was made to stratify the conditions by sex at birth. Participants completed the study between February and April 2023.

The study was conducted according to the revised Declaration of Helsinki (2011) and the 1996 International Council for Harmonisation (ICH) Guidelines for Good Clinical Practice E6 (R2). All participants provided informed consent online prior to taking part in the study and were compensated for their time. The study protocol was pre-registered on the Open Science Framework (OSF) https://doi.org/10.17605/OSF.IO/F95A2.

### Participants

1242 young adults were assessed for eligibility during the pre-screening phase. Inclusion criteria included a late or early chronotype (as determined by a score of 4–11 or 18–26 on the reduced Morningness-Eveningness Questionnaire (rMEQ)^15^), aged between 18 and 25, residing in the UK, English language fluency and normal or corrected-to-normal vision. Exclusion criteria included a current diagnosis of depression or any sleep disorder (determined via self-report), use of antidepressant medication, daily nicotine (smoking/vaping) or recreational drug use, drinking more than 35 alcoholic units per week and drinking more than 8 cups of caffeinated beverages a day. 208 eligible adults (109 late chronotypes and 99 early chronotypes) were recruited and successfully completed the main study.

### Questionnaires

At the start of the main study session, the following measures were collected; sleep quality (Pittsburgh Sleep Quality Index (PSQI)^16^), number of hours slept the night before, positive and negative affect (Positive And Negative Affect Schedule (PANAS)^17^) and consumption of alcohol, caffeine, nicotine and recreational drugs within 24 hours. A repeated measure of positive and negative affect (PANAS) was collected after CBM training. All questionnaires are described in full in online supplemental material 1.

10.1136/bmjment-2024-301045.supp1 Supplementary data

### Cognitive bias modification

All participants completed an active version of CBM training for facial emotion processing as described previously.^11 18^ Briefly, the task involved viewing 15 images of facial expressions morphing from happy to sad. In the baseline block, participants judged the emotion (ie, happy or sad, two-option forced choice) and a ‘balance point’ is calculated (interpretation bias towards ambiguous facial expressions). Next, participants underwent three blocks of active CBM training where participants were given feedback that the two faces adjacent to their balance point (training threshold) should be classed as ‘happy’ instead of ‘sad’, positively shifting responses to the faces (Supp figure 1). The baseline block was then repeated to assess the post-training balance point. Further task details are available in supplementary materials.

### Sample size determination

Previous studies of CBM versus sham in healthy individuals with low mood show a large effect of CBM training on the post-training balance point.^10^ Previous studies of synchrony effects on executive functions are large.^13^ Therefore, we cautiously powered the current study for the main effect of synchrony (2×2 analysis of variance (ANOVA)) on post-training balance point assuming a smaller effect size (Cohen’s f=0.2). A total sample of 200 (50 per group) gave us 80% power to detect this effect at an alpha rate of 0.05. We recruited an extra eight participants (total n=208) in error before the study was closed.

### Data cleaning

Data wrangling was done in R and analysed using IBM SPSS Statistics (V.29). Participants were excluded from analyses if: they failed to complete all CBM blocks, had an extreme baseline balance point (<4 or >12) or >2× the IQR away from the median, or completed the study >2 hours outside of their time slot. Participants were asked not to consume alcohol or recreational drugs within 24 hours, nicotine within 12 hours or caffeine within 1 hour of completing the study and were excluded from analysis if they reported they had (self-reported at the beginning of the study session).

### Computational model

An updated version of an ALCOVE computational model of associative learning^19^ was fitted to trial-by-trial responses during CBM training and was used to compute the primary outcome measure; effective learning rate (ε_effthr_). Model details are described in the Supplementary materials. Briefly, the effective learning rate is a measure of how quickly the participant incorporates task feedback to update their responses to new faces. People generalise what they learn about each face stimulus according to its relationship to neighbouring stimuli on the facial expression morph continuum. This is accounted for in the effective learning rate which adjusts for individual training threshold. Higher effective learning rates indicate the participants respond more quickly and effectively to task feedback and therefore shift their classifications of ambiguous faces accordingly. Other parameters included: (1) inverse temperature ( θ ) (response reliability), (2) emotion bias (gH – gS) (positive values represent excess sad judgements on overt facial expressions) and (3) pre-training indifference point (a model-based measure analogous to balance point).

### Statistical analyses

The primary outcome measure was effective learning rate (ε_effthr_) which was transformed to a normal distribution using an inverse rank transformation. The secondary outcome measure was the post-training balance point, calculated as: (the number of ‘happy’ responses in the post-training baseline block/45 trials)×15 facial stimuli. A 2×2 ANOVA tested for a main effect of group (late chronotype, early chronotype), condition (synchronous, asynchronous) and a group×condition interaction on the primary (learning rate) and secondary (post-training balance point) outcome measures. Exploratory analysis of covariance were also performed adjusting for pre-training outcome measures, negative affect symptoms (PANAS score) and sleep quality (PSQI score) by including these variables as covariates in the model. Pre-training outcome measures (eg, pre-training balance point) were included as covariates to adjust for the participant’s baseline (eg, this can be interpreted as the effect of group and condition on change in balance point). Sleep quality and negative affect were included as covariates due to the effects of poor sleep quality and higher negative effects on attention, cognition and the perception of negative facial expressions.^20^ Since age was within a narrow range^10 14 18–23^ and sex was balanced between conditions, these variables were not added as covariates. All analyses were conducted on eligible participants (n=173), however for completeness, analyses were also conducted on the full sample (including those who had used nicotine, alcohol, caffeine and recreational drugs close to the study, n=208). Exploratory analyses included post-training positive and negative affect (subscales of PANAS score) as the outcome variables. Further details in supplementary materials.

## Findings

### Quality control

All participants completed all CBM blocks. Two participants were excluded as they had balance points lower than 4. Three participants completed the session outside of their allotted time slot (due to experimenter error) but none >2 hours as per the quality control criteria. 34 participants were excluded for reporting using stimulants (alcohol, nicotine, recreational drugs, caffeine) before the study session leaving 173 eligible participants (47 late chronotype synchronous, 45 late chronotype asynchronous, 40 early chronotype synchronous, 41 early chronotype asynchronous).

### Participant characteristics

Baseline characteristics are presented in table 1. Across the sample (n=173), 114 reported their ethnicity as ‘White’, 20 as ‘Black’, 25 as ‘Asian’, 10 as ‘Mixed’ and 4 as ‘Other’. 77 were in full or part-time employment, 58 were studying full or part-time, 18 were unemployed and 20 were unknown. The average age was 22.5 years and 45% were men. Sleep quality (M=5.0, SD=2.3) was poor as some studies suggest that a PSQI score >5 represents sleep disturbance.^21^ Late chronotypes were significantly younger, had poorer sleep quality, slept fewer hours the night before the study session and had lower positive effect compared with early chronotypes. A larger proportion of late chronotypes were also men. The proportion of participants who used nicotine, alcohol, caffeine and recreational drugs did not differ between groups. The mean times that participants completed the study session within groups and conditions were at: 19:29 late chronotype synchronous, 09:08 late chronotype asynchronous, 08:35 early chronotype synchronous and 19:44 early chronotype asynchronous.

**Table 1:** Baseline characteristics

Baseline characteristics	Late chronotype (n=92)	Early chronotype (n=81)	Statistics
Age (18–25)	21.9 (2.1)	23.2 (2.0)	t(171)=−4.3, p<0.001
Sex (M/F)	51/41 (55%/45%)	27/54 (33%/67%)	X^2^(1, n=173)=8.5, p=0.004
Sleep quality (PSQI)	5.4 (2.4)	4.5 (2.2)	t(171)=2.7, p=0.007
Hours slept the previous night	7.3 (1.7)	7.7 (1.0)	t(171)=−2.0, p=0.05
Balance point	6.7 (1.3)	6.8 (1.2)	t(171)=−0.2, p=0.86
Positive affect (PANAS)	27.7 (6.9)	32.7 (9.3)	t(146.4)=−4.1, p<0.001
Negative affect (PANAS)	17.4 (6.3)	16.9 (6.0)	t(171)=0.6, p=0.55
Smoking/vaping status (yes/no)	7/85 (8%/92%)	4/77 (5%/95%)	X^2^(1, n=173)=0.52, p=0.55
Alcohol status (yes/no)	58/34 (63%/37%)	40/41 (49%/51%)	X^2^(1, n=173)=3.3, p=0.091
Caffeine status (yes/no)	69/23 (75%/25%)	55/26 (67%/33%)	X^2^(1, n=173)=1.1, p=0.32
Recreational drugs status (yes/no)	11/79 (12%/88%)	4/71 (5%/95%)	X^2^(1, n=167)=4.0, p=0.14
**Model parameters (median, IQR**)			
Effective learning rate (εeffthr)	0.14 (0.2)	0.15 (0.2)	t(171)=−0.12, p=0.90
Generalisation (σ)	6.2 (12.2)	3.5 (13.1)	t(171)=0.99, p=0.32
Emotion bias (gH − gS)	0 (0)	0 (0.04)	t(171)=−1.1, p=0.29
Inverse temperature (θ)	3.3 (8.2)	3.6 (8.1)	t(171)=−0.77, p=0.45
Indifference point	7.0 (1.6)	7.3 (1.2)	t(170)=−1.75, p=0.082

Note: Numbers represent means (M) and SDs of the sample except for the model parameters where the median and IQR are reported. PSQI: Pittsburgh Sleep Quality Index (score range=0–21 where high scores represent poorer sleep quality), PANAS: Positive And Negative Affect Schedule (score range=10–50 for both subscales). Independent-sample t-tests and χ^2^ tests were used to compare values between chronotype groups.

### Validation of computational model parameters

Participant effective learning rate and generalisation were consistent with a prior report^8^ (table 1). As expected, the pre-training indifference point was strongly correlated to the pre-training balance point (r(172=0.87, p<0.001).

### Validation of CBM training effect

**Figure 2:**
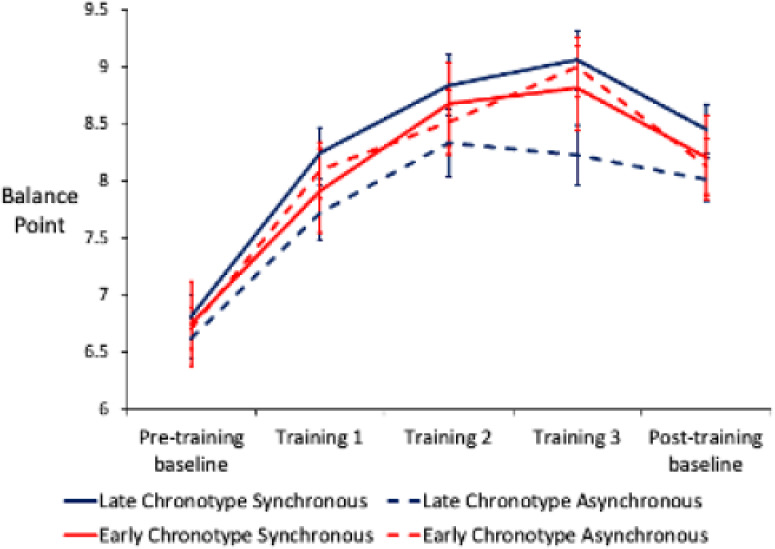
Shift in balance point from pre-training to post-training. The shift demonstrates a shift in categorisation of emotional facial expressions. Data points represent mean balance points with SE bars.

Overall, there was strong evidence that CBM produced positive changes in participant balance point rising from 6.75 (SD=1.24) at pre-training to 8.29 (SD=1.47) post-training (mean difference=1.52, SD=0.86, paired t-test t(172)=−22.1, p<0.001) (figure 2). This means that participants changed their responses to ambiguous facial expressions in line with the feedback they received. This positive change is in line with previous studies.^11^ However, there was an overall dip in balance point after training which could represent participants tiring of doing all blocks in a single session. Indeed, previous studies have asked participants to do five training blocks spread out over a week.^11^

### Effect of synchrony condition on effective learning rate (ε_effthr_)

**Figure 3:**
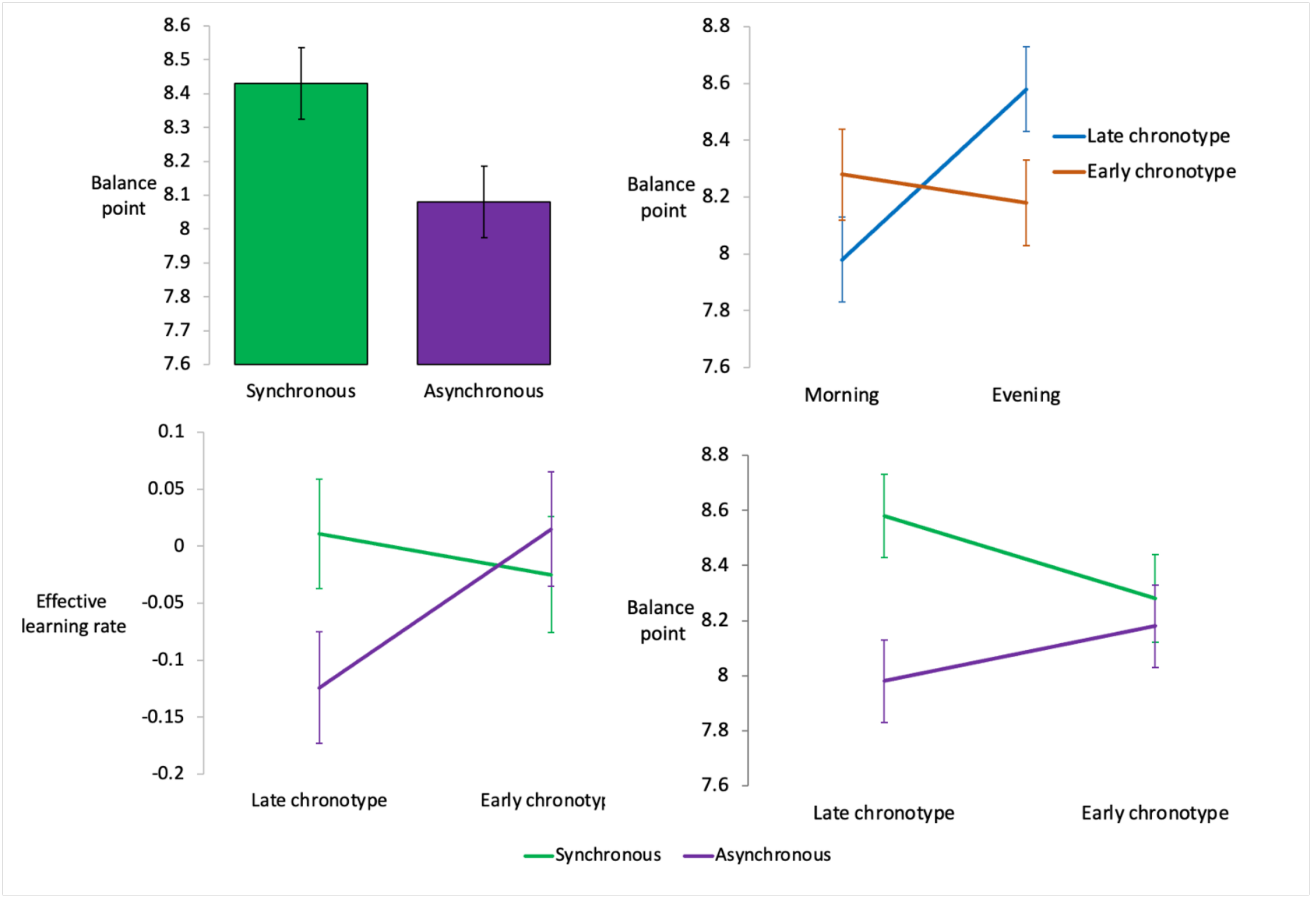
Main and interaction effects on measures of learning. (A) Bar chart showing participants were better able to shift their emotional perception of faces in the synchronous compared with asynchronous condition. (B) Line graph showing this effect split out by chronotype group where both chronotype groups perform better in the synchronous condition (morning for early chronotypes and evening for late chronotypes). (C) Line graph showing evidence that late chronotypes had a higher effective learning rate and (D) a higher post-training balance point in the synchronous (green lines) compared with asynchronous (purple lines) condition. Estimated values are adjusted for covariates.

There was no evidence for a main effect of the chronotype group, synchrony condition or interaction effect on the primary outcome measure; effective learning rate ε_effthr_. However, when sleep quality and pre-training negative affect were added as covariates to the model, there was weak evidence for a group×condition interaction effect (*F*(1,166)=2.92, p=0.090, η_p_^2^=0.017) where pairwise comparisons provided weak evidence that late chronotypes had a higher ε_effthr_ in the synchronous (M=0.012, SE=0.048) compared with asynchronous (M=−0.13, SE=0.051) condition (p=0.050) (figure 3C). This effect disappeared when participants who had recently taken stimulants were included in analyses.

### Effect of synchrony condition on balance point

There was no evidence for a main effect of the chronotype group on the secondary outcome measure; the post-training balance point. However, there was weak evidence for a main effect of synchrony (*F*(1,169)=3.40, p=0.067, η_p_^2^=0.020) where the post-training balance point was higher in the synchronous (M=8.50, SE=0.16) compared with the asynchronous (M=8.08, SE=0.16) condition. This effect was strengthened when sleep quality, pre-training balance point and negative affect were added as covariates (*F*(1,166)=4.28, p=0.040, η_p_^2^=0.025, synchronous condition M=8.43, SE=0.097, asynchronous condition M=8.15, SE=0.098) (figure 3A,B). There was moderate evidence for a group×condition interaction effect (*F*(1,166)=4.34, p=0.039, η_p_^2^=0.025) where pairwise comparisons (with Bonferroni correction) showed that late chronotypes had a higher post-training balance point in the synchronous (M=8.58, SE=0.13) compared with asynchronous (M=8.00, SE=0.14) condition (p=0.003) (figure 3D).

### Exploratory analyses

#### Effect of synchrony condition on positive and negative affect

Overall, there was strong evidence that CBM training decreased positive affect (mean difference=1.62, paired t-test t(173)=5.52, p<0.001) and negative affect (mean difference=1.59, paired t-test t(173)=6.51, p<0.001). When covariates were added, there was no evidence for a main effect of group, condition or interaction effect on positive affect but there was weak evidence for a main effect of condition on post-training negative affect where negative affect was higher in the synchronous (M=16.09, SE=0.33) compared with the asynchronous (M=15.16, SE=0.33) condition (*F*(1,168)=3.88, p=0.051, η_p_^2^=0.023). Further exploratory analyses are in supplementary materials.

## Discussion

This pilot study aimed to test whether the effectiveness of CBM for facial emotion processing is improved when delivered at a time-of-day that is synchronised to chronotype in healthy young adults. Overall, the learning rate did not show synchrony effects but there was a higher post-training balance point in the synchronous (evening for late chronotypes, morning for early chronotypes) compared with asynchronous (morning for late chronotypes, evening for early chronotypes) condition. This effect was strengthened when sleep quality, pre-training balance point and pre-training negative affect were controlled for. There was also weak to moderate evidence for an interaction effect where late chronotypes had a higher learning rate and post-training balance point during the synchronous compared with asynchronous conditions. While CBM decreased both positive and negative affect in all participants, negative affect was higher in the synchronous compared with asynchronous condition, contrary to our prediction. Our findings provisionally suggest that tailoring the delivery timing of this psychological intervention to a person’s chronotype may optimise its effectiveness, and this may be particularly important for late chronotypes.

Contrary to our prediction, the learning rate did not show synchrony effects. This suggests that the speed at which feedback was used to update judgements of emotional faces was not different between conditions. To our knowledge, no previous studies have investigated synchrony effects on learning rate, however, the decision-making literature shows individuals tend to take more risks^22^ and show poorer strategic reasoning^23^ at asynchronous compared with synchronous times-of-day. This finding could relate to our experimental design which involved three blocks of active training in one session. The data showed that participants incorporated feedback into their responses quickly (during the first training block) but this slowed towards the end when presumably participants tired.

As predicted, there was evidence of a synchrony effect on the post-training balance point. Circadian rhythms regulate alertness and arousal such that the timing of these rhythms determine physiological, intellectual and cognitive function.^14^ As far as we know, this is the first study investigating synchrony effects on a psychological intervention that involves viewing happy and sad emotional facial expressions, categorising the emotion and using feedback to make more positive judgements. This finding is in line with other studies showing synchrony effects on attention, working memory, decision-making and learning (but not facial processing)^24–26^—executive functions needed for this psychological intervention. It is noted that this synchrony effect was reported in participants free from alcohol, nicotine, recreational drugs and caffeine suggesting that stimulants that boost attention and cognitive performance may mask this effect.

As predicted, we showed that late chronotypes had a higher learning rate and post-training balance point in the synchronous compared with asynchronous condition (interaction effect). This provides initial evidence that late chronotypes are less able to adapt to completing CBM training at non-optimal times-of-day compared with early chronotypes. This supports a previous study showing that late chronotypes were less efficient (slower, less accurate and invested more cognitive resources) at solving an analogy detection task at a non-optimal time-of-day compared with early chronotypes.^27^ Late chronotypes also had reduced pre-experimental pupil baselines indicating poorer alertness and wakefulness.^27^ Indeed, late chronotypes in our sample had significantly poorer sleep quality and slept fewer hours the night before the experiment although both effects remained when sleep quality was adjusted for. This finding could be because late chronotypes experience more social jetlag (16.6% of young adults experience a delay of 2 hours^28^), although this was not directly measured. Interestingly, the learning rate also showed this interaction effect despite not being sensitive to an overall synchrony effect. Late chronotypes showed the poorest performance on CBM training in the asynchronous condition which could have meant that poor attention and engagement were accompanied by a poorer, or more impulsive, learning strategy.

Positive and negative affect scores were similar to previous samples^17^ and were both reduced after CBM training. Evidence for a therapeutic effect of CBM training in healthy^10^ and depressed^11^ samples is mixed. Here, we report a flattening towards a more neutral affective state. Exploratory analyses revealed that neither positive nor negative affect was improved differentially between chronotype groups. This could be due to our choice of scale (PANAS) which measures immediate changes in emotions, or that we did not do longer-term follow-ups,^11^ or be reflective of a non-clinical sample with low levels of negative affect. However, post-training negative affect was higher in the synchronous condition. This finding was unexpected, but previous (unpublished) data suggests that CBM training can have negative effects (eg, feeling frustrated by negative feedback) as well as positive. Negative effects may therefore have been amplified in the synchronous condition. If this result replicates, it may indicate individual differences in affective response to CBM training. Future studies would benefit from studying mood in clinical samples of depression with more robust measures of mood (eg, Immediate Mood Scaler) and follow-up measures.

### Limitations

First, the rMEQ is an indirect, behavioural measure of the circadian phase. Future studies would benefit from measuring other indices, for example, dim light melatonin onset. It would be interesting to investigate which aspects determining chronotype (eg, genetics, light exposure) are most important for synchrony effects. Second, the timings of CBM training sessions (morning/evening) were based around working hours and were generous (4-hour slots) meaning participants may have completed the session at different phases of their circadian physiology. Future studies would benefit from fixing the session to circadian time (eg, hours after waking). Third, all measures were self-reported and there were likely other factors, for example, light, activity, breakfast or naps affecting cognitive performance that were not accounted for. Future studies would benefit from controlled laboratory conditions (eg, to control stimulant use) to validate and extend this work.

### Clinical implications

Initial evidence is that an online psychological intervention can be optimised (learning is improved) when delivered at a time-of-day, that is, synchronised to chronotype, and this might be most important for late chronotypes that are vulnerable to poor mental health. This could be considered during intervention delivery. Chronotherapy has shown that medication delivery timing for physical and mental health conditions can improve treatment effects^29^ however there are concerns about whether treatments can be delivered at certain times and if circadian rhythms can be measured accurately enough. Interventions that can be delivered online could avoid some of these practical concerns. Future studies in clinical populations, for example, depression, would be important for understanding the effect on mood.

## Data Availability

The data and analysis code that form the basis of the results presented here are available from the University of Bristol’s Research Data Repository (http://data.bris.ac.uk), DOI: https://doi.org/10.5523/bris.1p8g48bwqgio52g7yb5a28g9xc
