# Supplementary material for: Chronotype and emotion processing: a pilot study testing timing of online cognitive bias modification training
Source: BMJ Ment Health. 2024 Jul 2;27(1):1–7. doi: 10.1136/bmjment-2024-301045 (PMC11227755; doi:10.1136/bmjment-2024-301045)
Supplement: Supplementary data [file bmjment-2024-301045supp001.pdf]

Supplementary Materials

Methods

Cognitive Bias Modification task details

Participants viewed 15 images of facial expressions morphing from happy to sad, as reported previously (I. S. Penton-Voak et al., 2021; Suddell et al., 2021). The faces ranged on a continuum with different levels of emotional intensity, from an unambiguous happy face to an unambiguous sad face. In the baseline measure of CBM, the participant was briefly shown these images one-by-one and asked to judge what emotion is shown on the face (i.e., happy or sad, two-option forced choice). Initially participants were not given feedback, allowing for a ‘balance point’ to be established. This refers to the image at which the participant is equally likely to judge the face as happy or sad, indicating the natural interpretation bias an individual holds towards ambiguous facial expressions. Next, participants underwent three blocks of active CBM training. Participants were given feedback on which facial stimuli were happy and which were sad (Figure 2B) during active training. This feedback was tailored to shift each participant’s unique balance point and was displayed on the screen after every response. The participant received feedback on the two faces adjacent to their individual balance point (rounded to nearest integer) which they previously categorised as ‘sad’ (training threshold). Participants received feedback that these faces should be classified as ‘happy’. This trained the participant to categorise and perceive these facial expressions more positively. After the CBM training, the baseline measure was repeated, and the participants no longer received any feedback. This was to assess the post-training balance point. A shift in the balance point demonstrates a change in the participants’ responses to emotional expressions (Supplementary Figure 1A).

There were 45 trials in the baseline measure, and each facial stimulus was randomly presented three times. Each training block contained 31 trials. Facial stimuli were randomly presented with a bias towards presenting the ambiguous faces three times and the unambiguous faces one-to-two times per training block. The fixation cross was displayed for 1500-2500ms and then the facial stimuli was presented for 150ms. Following this, a mask was presented for 250ms before a response window was displayed. Feedback in the training block was presented for 1000ms (Supplementary Figure 1B). An entire CBM session, including baseline and training, took up to 15 minutes to complete.

Overall, it was much easier to find and recruit late chronotypes to the study than early chronotypes.

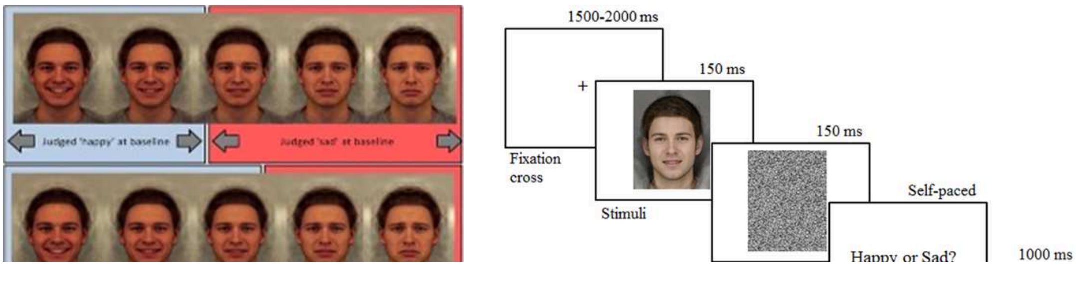

Supplementary Figure 1. Cognitive Bias Modification (CBM) training. A) In the baseline assessment, participants viewed 15 faces morphed between happy and sad. They judged these expressions to determine their 'balance point' (example in top panel). The training session provided tailored feedback after each trial to shift their balance point and emotion perception (example in bottom panel). B) Example CBM session training block with feedback presented. The baseline blocks followed an identical procedure but without the feedback window. Figure adapted from previous paper (*23*).

Questionnaires

Reduced Morningness Eveningness Questionnaire (rMEQ) – a 5-item measure of chronotype developed by Adan and Almirall (1991). The reduced version is based on the full 19-item MEQ version and has good reliability, validity and re-test reliability. Total scores range from 4-26 where higher scores indicate increased morningness and cut-off scores indicate chronotype groups: late chronotype <12, neither chronotype 12-17, early chronotype >17.

Pittsburgh Sleep Quality Index (PSQI) – a 19-item measure of sleep quality and disturbances over the past month (Buysse et al., 1989). Seven component scores assessing subjective sleep quality, sleep latency, sleep duration, habitual sleep efficiency, sleep disturbances, use of sleeping medication and daytime dysfunction are summed to create a global score ranging from 0-21. A score >5 indicates poor sleep quality.

Positive and Negative Affect Schedule (PANAS) – a 20-item measure including 10 items measuring positive affect and 10 items measuring negative affect (Watson et al., 1988). The scale is a measure of immediate emotions (e.g., hostility, excitedness) and was used in the current study in order to measure a change in immediate mood before and after CBM training.

Computational model

An updated version of an ALCOVE computational model of associative learning (*24*) was used to compute the primary outcome measure; learning rate. The model is fitted to trial-by-trial responses as detailed here. Briefly, the model uses a connectionist framework to model error-driven learning during the CBM task. The update to the model allows it to read in pre-training data to account for an individual's bias in judging overt and ambiguous happy and sad facial expressions (*25*). The model is parameterised to estimate a maximum effective learning rate (ε_{effmax}), referring to the greatest speed along the morph continuum in which feedback is incorporated into new responses. Learning applied to new judgments depends on the position the facial stimulus on the morph continuum, resulting in an effective learning rate at each morph. The maximum effective learning rate (ε_{effmax})

ranges from 0-1 where higher values represent a greater degree of the prediction error contributing to updating judgements of faces. The model also estimates generalisation (σ), the degree to which feedback from the prior trial is used to update responses to adjacent stimuli on the morph continuum. Here, the effective learning rate is adjusted to the training threshold (ε_{effthr}) i.e., there is no maximum limit and it is centred around the facial morphs that the participant was receiving feedback on. Other parameters included: 1) inverse temperature (θ) (where higher values represent increased response reliability), 2) emotion bias (gH – gS) (ranging between -1 to 1 where positive values represent excess sad judgements on overt facial expressions), and 3) the pretraining indifference point (a model-based measure of the point at which participants distinguish between happy and sad faces, analogous to the balance point).

Exploratory analyses

In an exploratory analysis, the outcome measures were the post-training positive and post-training negative affect (subscales of PANAS score). The change in affect from pre to post training (paired t-test) was tested and 2x2 ANOVAs were performed to examine whether changes in affect after CBM training interact with chronotype group and synchrony condition. Finally, additional parameters derived from the computational model of associative learning are reported and interpreted including: gH – gS bias, inverse temperature (θ) and generalisation (σ).

Results

Exploratory analyses

Positive affect

Overall, there was strong evidence that 3 blocks of active CBM produced changes in participant positive affect decreasing from 30.0 (SD = 8.5) to 28.4 (SD = 9.0) (mean difference = 1.62, paired t-test t(173) = 5.52, p<.001). There was strong evidence for a main effect of group on post-training positive affect where late chronotypes (M = 26.49, SE = .92) had lower positive affect compared to early chronotypes (M = 30.60, SE = .97) (F(1,170) = 9.43, p = .002, η_p^2 = .053). However, this effect disappeared when pre-training positive affect and sleep quality were added as covariates. There was no evidence for a main effect of condition or interaction effect. When participants that had recently taken stimulants were included in analyses, there was weak evidence for a main effect of condition where post-training positive affect was higher in the synchronous (M = 29.42, SE = .38) compared to asynchronous (M = 28.40, SE = .39) condition (F(1,201) = 3.5, p = .063, η_p^2 = .017).

Negative affect

Overall, there was also strong evidence that 3 blocks of active CBM produced changes in participant negative affect decreasing from 17.2 (SD = 6.2) to 15.6 (SD = 6.1) (mean difference = 1.59, paired t-test t(173) = 6.51, p<.001). There was no evidence for a main effect of group or condition on post-training negative affect. However, there was moderate evidence for a group x condition interaction where late chronotypes had higher post-training negative affect in the synchronous (M = 17.55, SE = .88) compared to asynchronous (M = 14.07, SE = .90) condition (F(1,170) = 5.77, p = .017, η_p^2 = .033). However, this effect disappeared when pre-training negative affect and sleep quality were added as covariates to the model. Instead, there was weak evidence for a main effect of condition on post-training negative affect where negative affect was higher in the synchronous (M = 16.09, SE = .33) compared to the asynchronous (M = 15.16, SE = .33) condition (F(1,168) = 3.88, p = .051, η_p^2 = .023). Weak evidence for this effect remained when participants that had recently taken stimulants were included in analyses (synchronous M = 16.29, SE = .30, asynchronous M = 15.52, SE = .30, F(1,201) = 3.29, p = .071, η_p^2 = .016).

Additional model parameters

There was no evidence for a main effect of chronotype group or synchrony condition on emotion bias (gH – gS) or generalisation (σ), or any group x condition interactions on these parameters. This did not change when sleep quality and negative affect were added as covariates, or when the full sample was included.
